# Computer simulations of coupled idiosyncrasies in speech perception and speech production with COSMO, a perceptuo-motor Bayesian model of speech communication

**DOI:** 10.1371/journal.pone.0210302

**Published:** 2019-01-11

**Authors:** Marie-Lou Barnaud, Jean-Luc Schwartz, Pierre Bessière, Julien Diard

**Affiliations:** 1 Univ. Grenoble Alpes, Gipsa-lab, Grenoble, France; 2 CNRS, Gipsa-lab, Grenoble, France; 3 Univ. Grenoble Alpes, LPNC, Grenoble, France; 4 CNRS, LPNC, Grenoble, France; 5 CNRS - SORBONNE Université - ISIR, Paris, France; Universitat Potsdam, GERMANY

## Abstract

The existence of a functional relationship between speech perception and production systems is now widely accepted, but the exact nature and role of this relationship remains quite unclear. The existence of idiosyncrasies in production and in perception sheds interesting light on the nature of the link. Indeed, a number of studies explore inter-individual variability in auditory and motor prototypes within a given language, and provide evidence for a link between both sets. In this paper, we attempt to simulate one study on coupled idiosyncrasies in the perception and production of French oral vowels, within COSMO, a Bayesian computational model of speech communication. First, we show that if the learning process in COSMO includes a communicative mechanism between a Learning Agent and a Master Agent, vowel production does display idiosyncrasies. Second, we implement within COSMO three models for speech perception that are, respectively, auditory, motor and perceptuo-motor. We show that no idiosyncrasy in perception can be obtained in the auditory model, since it is optimally tuned to the learning environment, which does not include the motor variability of the Learning Agent. On the contrary, motor and perceptuo-motor models provide perception idiosyncrasies correlated with idiosyncrasies in production. We draw conclusions about the role and importance of motor processes in speech perception, and propose a perceptuo-motor model in which auditory processing would enable optimal processing of learned sounds and motor processing would be helpful in unlearned adverse conditions.

## Introduction

### Auditory vs. articulatory invariance in speech communication

The nature of phonetic invariance has been at the heart of lively exchanges among speech communication researchers [1]. The debate was first focused on speech perception, opposing tenants of auditory theories and auditory invariants [2, 3] and proponents of the motor theory of speech perception claiming that phonetic invariants would be motor [4, 5]; or rather articulatory in the direct realist perspective [6]. The debate propagated to the domain of speech production, once again contrasting the view that motor targets would be specified in terms of articulatory constrictions and vocal tract shapes [7, 8] and arguments in favor of auditory targets as reference frames for programming speech gestures [9–12].

Progress in neurocognitive techniques and paradigms has strongly strengthened the experimental bases of this debate in the last twenty years and made it clear that perceptuo-motor interactions shape both sides of the speech communication process. The role of auditory representations in speech production appears crucial, not only for learning phonetic targets, but also for adapting and compensating for perturbations [13–15]. On the flip side, neurophysiological and behavioral experimental data have also shown more and more clearly that both auditory and motor systems are activated during speech perception [16–18]. A converging set of recent studies confirms that the speech production system is activated and has a significant role in the speech perception process, particularly in adverse conditions [19–32]. Globally, it appears that, in the human brain, “the hearing ear is always found close to the speaking tongue”, quoting part of the title of a recent paper by [33].

The key aim is to really understand the role of the “hearing ear” when the tongue speaks, and the role of the “speaking tongue” when the ear hears. In other words, our overall goal is to assess how speech perception processes intervene in speech production and, conversely, how speech production knowledge constrains and informs speech perception.

### Perceptuo-motor idiosyncrasies as a test case for the proximity of the speaking tongue and the hearing ear

While the precise understanding of invariants associated to each and every phonetic category is probably currently out of reach, the way variability is structured along situations and contexts may be quite enlightening about the relationships between speech production and speech perception. Variability associated to coarticulation, with the sound of a given phoneme depending on the neighboring phonemes, actually provided the main push for arguing in favor of the possible existence of articulatory or motor invariance [4, 6]. Variability in response to sensory or motor perturbations provided evidence for the role of sensory knowledge in speech production [13–15] or motor knowledge in speech perception [34, 35].

Another important source of variation comes from inter-subject variability. Part of such variability just results from physical differences in size and shape of the vocal tract related to sex, age and morphology, or associated to handicaps or the consequence of clinical operations. Another part results from variations in the subject’s communication environment, modifying the learning process and consequently producing dialectal or sociolinguistic variation. But even factoring out these extrinsic causes of inter-individual variation, we will see that there remains a part of phonetic variation, which appears as “free”, that is to say, resulting from phonetic specificity of individual speakers. This is what we refer to in the following as *free idiosyncrasies*.

The fact that part of inter-individual variability is indeed free rather than constrained by the physical, biological, linguistic or sociocultural environment is best evidenced by studies of speech produced by members of the same family. A number of studies have been published concerning monozygotic or dizygotic twin pairs, providing both similarities and differences in vocal tract and vocal source characteristics [36–38]. In a recent study on vowel production in 10 pairs of French adult male siblings, [39] showed that the inter-individual acoustic variability was globally lower between brothers, compared with speakers that were not brothers. However, the sibling factor explained only a small part of the global inter-individual variability, confirming the free nature of idiosyncrasies.

Interestingly, idiosyncrasies may also be observed in speech perception, as revealed by the specific manner in which a listener categorizes an acoustic space. Moreover, a small number of studies have attempted to assess whether there was a link between idiosyncrasies in perception and production. It actually appears to be the case, as shown by studies on vowels [40, 41] or stop consonant voicing or place [42]. In a recent paper, [43] analyzed the production and perception of oral French vowels by twelve subjects. They focused their analysis on the inter-individual variations of the first formant (F1) in relation to the four degrees of vowel height in the French oral vowel system. All values were normalized in frequency in order to reduce variability related to differences in sex, age and size (see later for more details on the protocol).

Their results show that, while F1 values display a clear trend to be stable among front unrounded, front rounded and back rounded vowels for a given degree of vowel height, the F1 values differ substantially from one subject to another. Such idiosyncrasies are observed both in perception and in production, that is, subjects differ both in their production of vowels and in the acoustic values providing the center of their perceptual categories in a speech perception experiment (see Section “The Ménard & Schwartz study [43]” for more details). Strikingly, [43] found strong correlations between the perceived and the produced F1 values among the 12 subjects for each tested vowel. Therefore, part of the subjects’ variability in production does appear as free rather than guided by the learning environment, and it is associated to a correlated variability in perception. This suggests that in speech idiosyncrasies also, the hearing ear is close to the speaking tongue.

### Modeling perceptuo-motor idiosyncrasies to better understand perceptuo-motor relationships in speech communication

Free idiosyncrasies suggest that the development of speech production is guided not only by the data provided by the environment, but also by specific adaptations in which the speaker selects strategies that she/he considers adequate for communication. Conversely, the fact that idiosyncrasies in perception mirror idiosyncrasies in production suggests that speech production knowledge might intervene in speech perception. Computational models are crucial tools to attempt to better assess and understand this potential bidirectional coupling between perception and action.

#### Idiosyncrasies in production

There exist several computational models of speech production, differing, among other points, on the nature of the target for motor commands. The “articulatory” conception theorized in articulatory phonology [7, 8] led to the development of the Task Dynamics model [44] in which the coordination of articulators described in a dynamic framework leads to a sequence of articulatory configurations (defined by constriction place and size) along a gestural score. Conversely, motor coordination in auditory models of speech production such as DIVA (Directions Into Velocities of Articulators; [10, 45]) or GEPPETO (GEstures shaped by the Physics and by a PErceptually oriented Targets Optimization; [12, 46] is driven by a sequence of auditory objectives defined as target areas in the auditory space (typically the formant space). The state feedback control model developed by [47] further describes how the speech motor system could then dynamically integrate sensory information within motor commands.

However, the existence of free idiosyncrasies in production has seldom been studied in computational models. It raises interesting questions about the way variability may result from the learning process. Developmental models of speech acquisition classically incorporate a process similar to imitation in which the computational learning agent attempts to reproduce the sounds of its environment [10, 48, 49]. The first assumption that will be explored in this paper is that the generation of idiosyncrasies should lead to replace an “imitative process” by a “communicative” process. In the first case, the development of the speech production process requires the learning agent to attempt to precisely reproduce the sound produced by a “master”, while in the second case, the learning agent rather attempts to produce acoustic stimuli enabling reproduction of the phonological target, even though the produced sound may differ from the exact sound provided by the master. Therefore, idiosyncrasies in speech production would be based on the possibility for different agents to explore different areas in the target region defined in the auditory space. This first hypothesis hence addresses the way the hearing ear should guide the speaking tongue in the course of speech production development within a communicative process.

#### Idiosyncrasies in perception

In the field of speech perception, there are a number of computational models of auditory theories. To take a recent example, the “Ideal Adapter” model [50] can be considered a typical specimen of an auditory theory, in which speech perception is considered a Bayesian inference process operating on a model of the auditory environment [51, 52]. Conversely, computational models of the motor theory are badly lacking, in large part because of the difficulty to solve the acoustic-to-articulatory inversion process necessary to infer articulatory or motor configurations from acoustic inputs [53]. Recent work by [54] and [55], which focus on the role of motor representations in the cognitive processing of speech, particularly in noisy environments, can be considered frameworks for modeling motor theories (see also the developmental models of the emergence of phonetic features by [56]).

However, perceptual idiosyncrasies have never, to our knowledge, been studied using computational models. The second hypothesis that will be explored in this paper is that the existence of perceptual idiosyncrasies coupled to motor idiosyncrasies suggests a link between motor and perceptual knowledge in the course of speech perception. The reasoning is that an auditory model of speech perception is based on the learning of the distribution of incoming speech sounds in the environment. In such a model, if several individuals are exposed in their development to the same set of sounds characterized by the same distribution, they should display no free idiosyncrasy in perception. Conversely, if there is some link between motor prototypes and auditory prototypes, free motor idiosyncrasies should result in coupled perceptual idiosyncrasies. This second hypothesis hence addresses the way the speaking tongue informs the hearing ear in the course of speech perception.

#### COSMO, a computational model for assessing the role of perceptuo-motor coupling in speech communication

Rigorous comparisons of auditory, motor and perceptuo-motor theories in a single modeling framework have, to the best of our knowledge, never been attempted systematically. This is why we have developed COSMO (for “Communicating Objects through Sensori-Motor Operations”), a computational model of speech communication enabling simulation, in a unified framework, of auditory, motor and perceptuo-motor theories of speech perception and speech production [57, 58]. In this model, which will be presented in detail later, a key component consists in a probabilistic link between sensory (*S*) and motor (*M*) variables, represented by a probability distribution *P*(*S* | *M*). This probabilistic “internal forward model”, supposed to be learned from the environment, provides the crucial link between auditory and motor representations, enabling computation of one from the other in a mathematically constrained way, both in the direct way (from *M* to *S*) and in the inverse way (from *S* to *M*), using Bayes theorem. By activating the sensory-motor link, perceptual information can be provided to the speech production system in the course of learning or online execution, and conversely, motor knowledge can feed decoding processes in the course of online speech perception.

In the COSMO framework, the implementation of a computational process associating motor inputs to sensory representations or sensory inputs to motor representations was the basic piece of knowledge that enabled us to analyze experimental data and relate them to theoretical predictions. In the present study, COSMO will be taken as a computational support to explore the two hypotheses introduced in the previous sections. For this aim, we will focus on the data about correlated auditory and motor idiosyncrasies observed by [43]. We will first assess how an imitative vs. a communicative learning process in COSMO may or may not lead to variations in motor commands resulting in idiosyncrasies in speech production (in relation to our first hypothesis). Then we will compare COSMO simulations of auditory, motor and perceptuo-motor theories of speech perception to assess in which conditions correlations between idiosyncrasies in speech perception and speech production might emerge (in relation to our second hypothesis).

The remaining of this paper is structured as follows. In Section “Simulation framework” dealing with the simulation environment, we first introduce the COSMO model, the way it is related to auditory, motor and perceptuo-motor theories of speech production and perception, and the implementation of the imitation vs. communication learning processes. Then, we present in some detail the experimental study by [43], which we will note “M&S” in the following, and which further provides the target for the simulations. Finally, we describe our experimental simulations and the evaluation tools. Section “Results” provides the simulations results before a discussion, in Section “Discussion”, about the nature of perceptuo-motor interactions for the characterization of phonetic units in speech communication.

## Simulation framework

### COSMO

#### Overview

The COSMO model is based on the analysis of a speech communication process, which can be described in the following way: a Speaker Agent utters linguistic objects (e.g., words) by activating its motor gestures. These gestures are received by a Listener Agent as sensory information and are decoded as linguistic objects. The communication is a success when both produced and perceived linguistic objects are identical.

We assume that the communication situation is internalized in an Agent composed of (i) a motor system associating motor representations with linguistic objects, (ii) a sensory system associating sensory representations with linguistic objects, (iii) a sensory-motor system associating motor and sensory representations, (iv) a validation system ensuring the coherence between linguistic objects in the motor and the sensory system. COSMO (“Communicating Objects using Sensory-Motor Operations”) is a probabilistic model implementing the relationships between all these variables, and exploiting these relationships for performing speech perception and production tasks.

Written in the Bayesian Programming framework [59, 60], COSMO encodes in a probabilistic manner the internal representations of the agent. It has already been defined, implemented and evaluated in previous work: to account for the emergence of sound systems in human languages [58], to demonstrate the indistinguishability between auditory and motor systems under “perfect learning conditions” [57, 61] and to analyze the complementary role of the auditory and motor systems [62, 63], in relation to neurocognitive data [64]. It has also provided a framework for the development of speech production models, analyzing the way they could deal with variability due to coarticulation [46] and adaptation to sensory perturbations [65].

#### Variables and distributions

**Fig 1:**
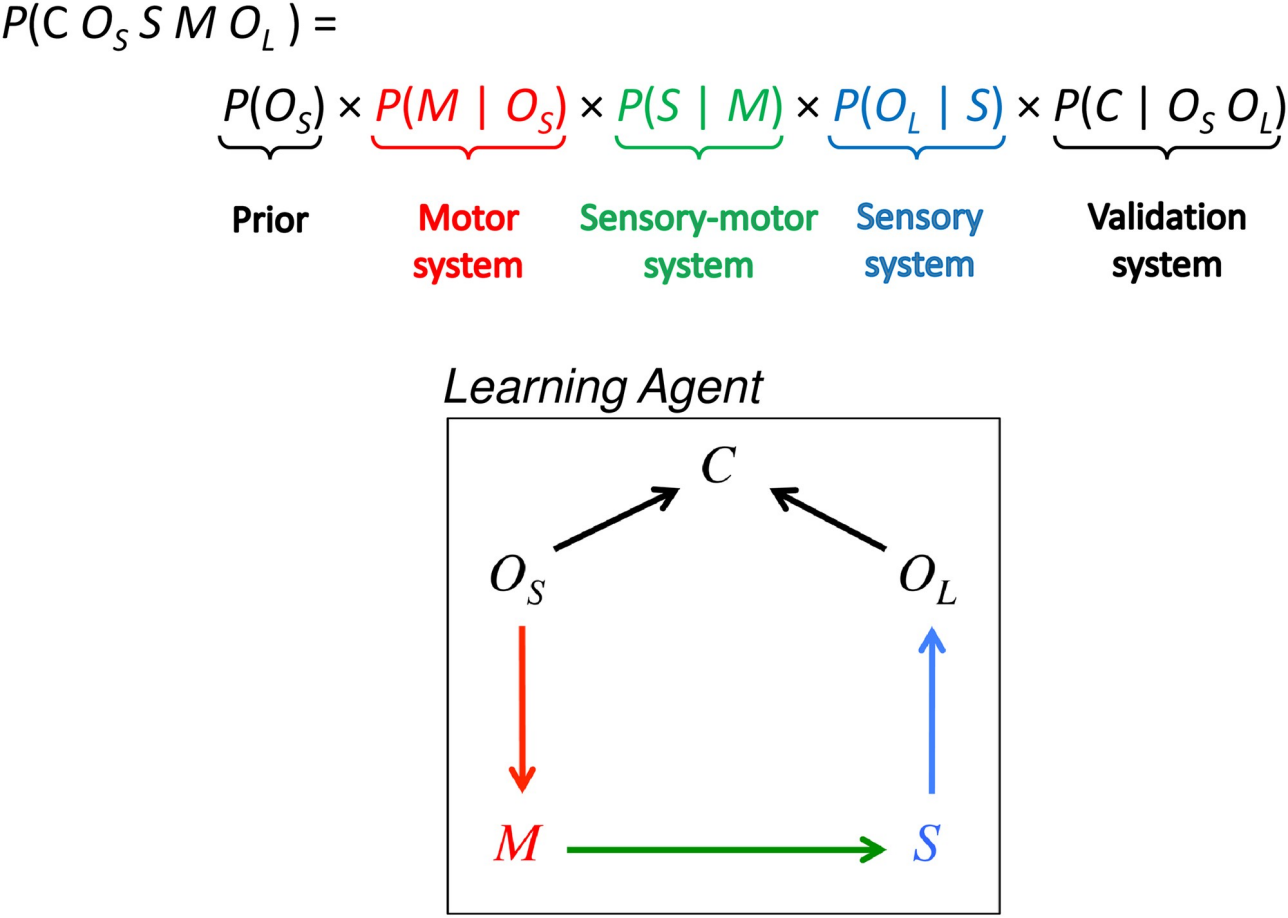
Decomposition of the joint equation of the COSMO model and the corresponding graphical illustration of the probabilistic dependency structure between variables. *C*: communication success, *O*_*S*_ and *O*_*L*_: linguistic objects, *M*: motor representation, *S*: sensory representation.

The COSMO model includes five probabilistic variables, each corresponding to an internalized representational space involved in the speech communication process, with a high level of abstraction: the linguistic objects *O*_*S*_ and *O*_*L*_ (respectively for the Speaker and the Listener), motor representations *M*, sensory representations *S* and, finally, a variable representing communication success, *C* (see Fig 1). Notice that there are two variables describing linguistic objects: one, *O*_*S*_ (the “object for the speaker”), is linked to motor representations *M* and the other, *O*_*L*_ (the “object for the listener”), is linked to sensory representations *S*. In the following of this paper, “objects” will refer to phonological units, typically vowel phonemes.

The five probabilistic variables of the COSMO model form a joint space over which a joint probability distribution *P*(*C O*_*S*_ *S M O*_*L*_) is defined. It is decomposed as a product of factors as follows [57]:

$$\begin{array}{l} P\left(C\;O_{S}\;S\;M\;O_{L}\right)\\ =P\left(O_{S}\right)P(M\;|\;O_{S})P(S\;|\;M)\\ P(O_{L}\;|\;S)P(C\;|\;O_{S}\;O_{L})\;. \end{array}$$ (1)

The probability distribution *P*(*O*_*S*_) encodes prior knowledge about the distribution of objects. The probability distribution *P*(*M* | *O*_*S*_) represents the motor system, giving probability distributions about motor gestures for each object *O*_*S*_. The distribution *P*(*S* | *M*) represents the sensory-motor system, linking motor gestures to the corresponding sensory values. The probability distribution *P*(*O*_*L*_ | *S*) represents the sensory system, associating sensory values with objects. Finally, the probability distribution *P*(*C* | *O*_*S*_ *O*_*L*_) ensures that the objects *O*_*S*_ and *O*_*L*_ are the same in the motor and sensory systems: *P*([*C* = 1] | *O*_*S*_ *O*_*L*_) is 1 if and only if *O*_*S*_ and *O*_*L*_ are identical, that is, they have the same value *o*. *C* is said to be a coherence variable and is used here as a “Bayesian switch” [66]. In the remaining of the paper, we use upper case characters for probabilistic variables and lower case characters for their values.

#### Learning

The probability distributions involved in Eq (1) must then be quantitatively specified so that the COSMO agent will be able to perceive and produce speech. The distribution *P*(*O*_*S*_) is generally considered uniform in our simulations, assuming that there is no prior knowledge on the probability of phonemes. The distribution *P*(*C* | *O*_*S*_ *O*_*L*_) follows the usual definition of Bayesian switches based on coherence variables. Therefore, three distributions remain to be specified: the sensory system *P*(*O*_*L*_ | *S*), the sensory-motor system *P*(*S* | *M*) and the motor system *P*(*M* | *O*_*S*_). In the present work, learning is assumed to be decomposed into three successive steps, sensory learning, sensory-motor learning and motor learning, in agreement with the developmental schedule proposed in the literature [67, 68]. Each learning phase exploits data provided by a Master Agent, that is, an agent that already has precise knowledge about the whole speech communication process. In this study, we consider that a Learning Agent has only one single Master, for the sake of simplification. We will comment in the Section Discussion the possible consequences of this simplification process.

**Sensory Learning** Sensory learning consists in characterizing the probabilistic relationship between phonemes and sounds. Technically, this is represented in COSMO by a “sensory repertoire” defined by distributions *P*(*S* | *O*_*L*_) that are learned from pairs of sounds and objects provided by the Master Agent. During each learning step, the Master Agent uniformly selects an object *o*. It produces a motor gesture by drawing in the distribution characterizing its production system. The drawn motor gesture results in a sound propagated in the environment and received by the Learning Agent as a stimulus sound *s*. We assume that the Learning Agent is able to both perceive and process the sound and directly recognize the object by some external information, provided by context. Therefore, learning is totally supervised.

In summary, the Master Agent provides pairs 〈*o*, *s*〉 and sensory learning results in updating parameters of the sensory system *P*(*S* | *O*_*L*_) in which [*O*_*L*_ = *o*].

**Sensory-motor Learning** Sensory-motor learning consists in characterizing the probabilistic relationship between gestures and sounds, represented in COSMO by the distribution *P*(*S* | *M*). Updating parameters of this distribution is more complex than for sensory learning, since the Master Agent cannot directly provide the produced motor configuration to the Learning Agent. In our model, this learning step involves an accommodation process. From the reception of a sound *s* produced by the Master Agent, the Learning Agent infers a motor gesture. It hence draws a motor gesture *m* according to the distribution *P*(*M* | [*S* = *s*]). The motor gesture *m* results in sound *s*′ generally different from the sound *s* uttered by the Master. The Learning Agent updates parameters of its sensory-motor system *P*(*S* | *M*) from the couple 〈*s*′, *m*〉.

**Motor Learning** Finally, motor learning consists in characterizing the probabilistic relationship between gestures and objects, that is, the distribution *P*(*M* | *O*_*S*_). To learn its motor system, the Learning Agent capitalizes on the previously learned systems, assuming that motor learning occurs at a later stage in development.

**Fig 2:**
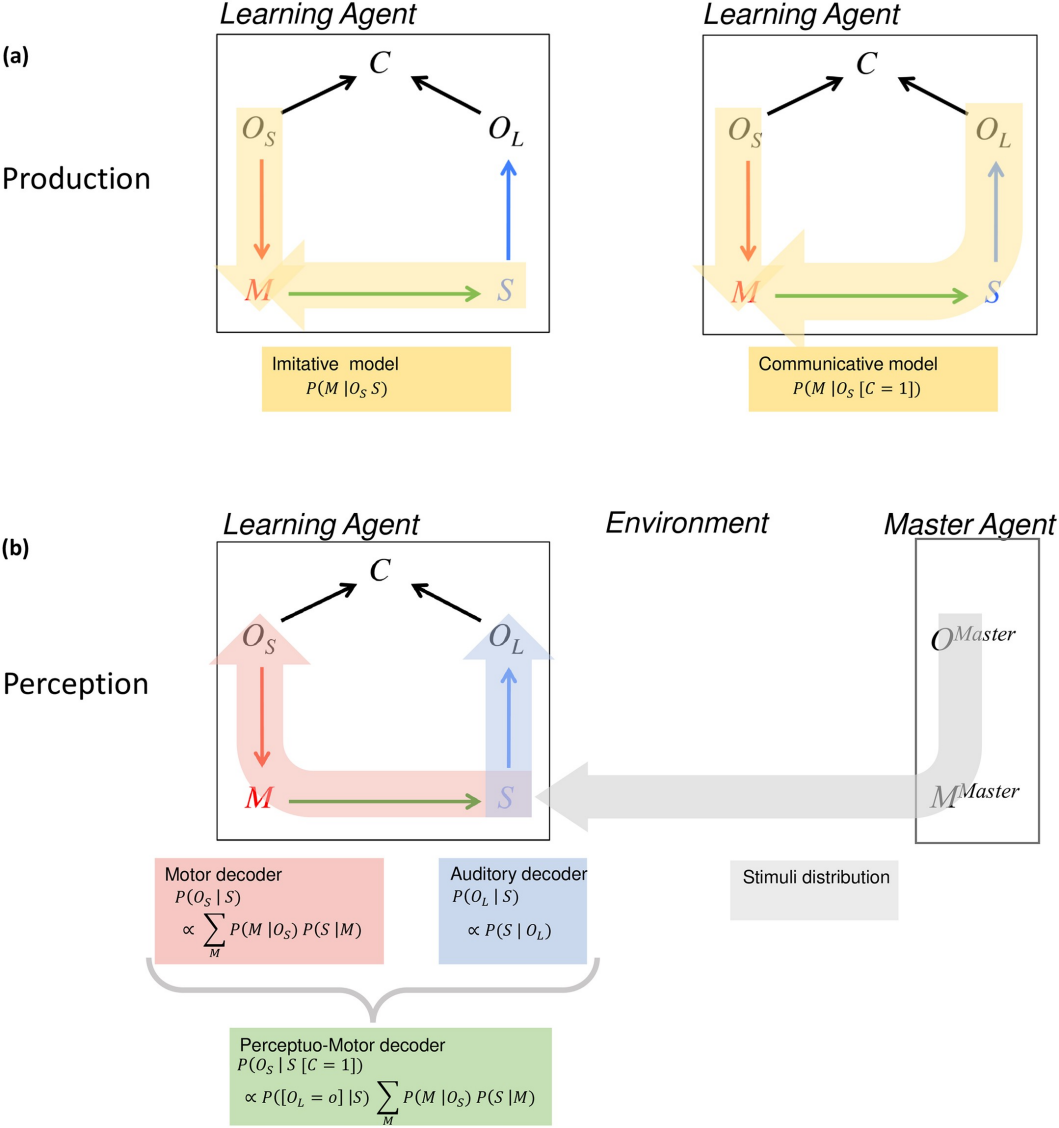
(a) Illustration of both motor learning models in COSMO: imitative model (left) and communicative model (right). (b) Illustration of the three perception theories in COSMO.

As in the learning of the sensory repertoire (sensory learning), we assume once again that the Master Agent provides pairs 〈*o*, *s*〉, that is an object and a corresponding sound. We also assume that the Learning Agent recognizes directly the object by means of contextual information, hence we stay in a supervised process in which [*O*_*S*_ = *o*]. To learn the *P*(*M* | *O*_*S*_) distribution, we introduce two competing models, together with the first working hypothesis of this study (see Fig 2a).

In the first motor learning model, that we call “imitative”, the Learning Agent attempts to reproduce the sounds provided by the Master Agent for a given object, by drawing upon the distribution *P*(*M* | [*O*_*S*_ = *o*][*S* = *s*]). In the second motor learning model, that we call “communicative”, the Learning Agent attempts to produce a sound likely to be understood by the Master Agent, by drawing a motor gesture according to the distribution *P*(*M* | [*O*_*S*_ = *o*][*C* = 1]). The [*C* = 1] condition means that the Agent not only associates the motor gesture *m* with the object *o* for the speaker, but also aims at being understood, hence at producing a sound that can be correctly decoded as the correct object *o*_*L*_ by the listener. Motor learning is hence based here on a communicative process. Then, the Agent produces *m* and the Master Agent receives the resulting sound *s*. If the Master Agent identifies the object corresponding to sound *s* as the initial *o*, the Learning Agent updates parameters of its motor system *P*(*M* | *O*_*S*_) with the couple 〈*m*, *o*〉.

The first hypothesis of this study is that free idiosyncrasies will be observed in the case of motor learning based on a communicative process, but not in the case of motor learning based on an imitative process. Idiosyncrasies, here, refer to variations in the acoustic realizations of a given phoneme, and not variations in the individual motor strategies associated to the production of a given sound. Notice furthermore that we focus here on free idiosyncrasies that are idiosyncrasies not induced by the environment, but rather related to a set of free variations in the Learning Agent in spite of the use of a fixed Master Agent. These two points will be further commented on in Section “Discussion”.

#### Implementing auditory, motor and perceptuo-motor theories in COSMO

Any behavioral task related to the COSMO model can be characterized by a probabilistic question addressed to the COSMO distribution in Eq (1), of the form *P*(*X* | *Y*), *X* and *Y* being subsets of the five COSMO variables *S*, *M*, *O*_*S*_, *O*_*L*_ and *C*. More precisely, speech production tasks in which a speaker would attempt at producing a gesture *M* adequate for a given phoneme *O* are formalized by questions of the form *P*(*M* | *O*). Conversely, speech perception tasks in which a listener would attempt at categorizing a phoneme *O* in response to a given stimulus *S* are formalized by questions of the form *P*(*O* | *S*).

However, these questions are not completely specified yet, since variable *O* in these equations could refer either to *O*_*S*_ or to *O*_*L*_. This is where a key assumption is introduced. It is considered that if *O* refers to *O*_*L*_, the listener is taken as the pivot for computations, which would correspond to simulating auditory theories. If *O* refers instead to *O*_*S*_, the speaker is taken as the pivot for computations, which would correspond to simulating motor theories. Finally, both *O*_*S*_ and *O*_*L*_ can simultaneously be associated to *O*, provided that the coherence variable *C* is set to 1: in this case, both the listener and the speaker would be involved in the computations, which would correspond to simulating perceptuo-motor theories (see Fig 2b).

Let us make clearer why the choices of *O*_*L*_ and *O*_*S*_ as the pivots in the computation do provide a likely framework for simulating auditory and motor theories, respectively. We will restrict this analysis here for speech perception theories, but the same could be done for speech production theories [57, 58].

In COSMO, an auditory theory would consist in associating a phonetic decoding task to the probability distribution *P*(*O*_*L*_ | *S*). This distribution is directly featured in Eq (1) as the “sensory system”. Practically, as we saw in the previous section, the COSMO implementation consists in assuming that the model learns a set of sensory prototypes (or a sensory repertoire) as a set of distributions *P*(*S* | *O*_*L*_). These distributions provide the statistics of sensory inputs for each category. Coming back to the distributions in the Learning Agent introduced in the previous section, decoding would then just consist in a Bayesian inversion of these sensory distributions:

$$\begin{array}{c} P\left(\left[O_{L}=o_{i}\right]\;|\;S\right)=\frac{P(S\;|\;\left[O_{L}=o_{i}\right])}{\sum_{j}P\left(S\;|\;\left[O_{L}=o_{j}\right]\right)}\;. \end{array}$$ (2)

This means that for a given input, all sensory distributions *P*(*S* | [*O*_*L*_ = *o*_*j*_]) are explored and the best—in the sense of Eq (2)—is selected. This computational model does fit naturally inside the definition of the “General Auditory and Learning Approaches to speech perception” introduced by [2], that they call the “General Approach” (GA) and define in the following way (p. 154): “GA does not invoke special mechanisms or modules to explain speech perception. Rather, it assumes, as a working hypothesis, that speech sounds are perceived using the same mechanisms of audition and perceptual learning that have evolved in humans or human ancestors to handle other classes of environmental sounds”.

Let us now consider how COSMO models a motor theory of speech perception. Motor decoding is characterized by the distribution *P*(*O*_*S*_ | *S*). This means that the COSMO model would search for a phonetic unit most likely to have produced a gesture resulting in the sound *S*. This is literally quite close to the “revised” version of the motor theory, in the classical paper by [5]. Quoting the abstract of this paper, phonetic perception would consist in detecting “the intended gestures of the speaker that are the basis for phonetic categories. Built into the structure of this module is the unique but lawful relationship between the gestures and the acoustic patterns in which they are variously overlapped”. Hence the theory states that the relationship between sounds and gestures enables the recovery of gestures from sounds, and gestures provide the basis for categorization. Technically, in COSMO, from the use of Eq (1) and principles of Bayesian inference, it can be shown that:

$$\begin{array}{l} P(O_{S}\;|\;S)\\ \;\propto \;\sum_{M}P(M\;|\;O_{S})P(S\;|\;M)\;. \end{array}$$ (3)

In the second member of this equation, the second factor *P*(*S* | *M*) conveys the “lawful relationship between the gestures and the acoustic patterns”, while the first factor *P*(*M* | *O*_*S*_) conveys the relationship between gestures and phonemes, since “the intended gestures of the speaker […] are the basis for phonetic categories”. While no computational model has ever been proposed by tenants of the Motor Theory to quantitatively simulate the theory’s principles, a number of algorithms have been introduced to deal with “articulatory-to-acoustic inversion” and motor decoding (see a review in [63]). Rather than selecting a single gesture from sound, Eq (3) expresses the Bayesian implementation of the inversion-and-motor-decoding process using a summation over all motor configurations weighted by the adequacy with the sound *S*—by *P*(*S* | *M*)—and inference of the phoneme *O*_*S*_—by *P*(*M* | *O*_*S*_).

This motor-to-sensory link is supposed to be innate in the motor theory (“This link, we argue, is not a learned association, a result of the fact that what people hear when they listen to speech is what they do when they speak. Rather, the link is innately specified, requiring only epigenetic development to bring it into play”, p. 3, [5]). However, no study previously proposed how such an innate link could be implemented. In the COSMO framework, the link is indeed learned in a precise way (see Section “Sensory-motor Learning”), and it provides a probabilistic implementation of the “Analysis-by-Synthesis” process introduced long ago by [69] and later developed by [70] and [33, 71] (see also [72] for a critical review). The fact that it is learned in COSMO rather than innate, as in the core of the motor theory, should hence not be considered limitation in our approach. As a matter of fact, it could be advocated that parts of learning processes have been at some stage encapsulated in innate components in the human brain. Still, the key point of our approach is to make computations explicit, in order to analyze contents of the computed representations and to interpret the differences between model variants in reference to theoretical proposals and experimental data.

Finally, the COSMO simulation of a perceptuo-motor theory of speech perception exploits the distribution *P*(*O*_*S*_ | *S* [*C* = 1]) and Bayesian inference leads to:

$$\begin{array}{l} P(\left[O=o\right]\;|\;S\;\left[C=1\right])\\ \;\propto \;P(\left[O_{L}=o\right]\;|\;S)\\ \;\;\sum_{M}\left(P(M\;|\;\left[O_{S}=o\right])P(S\;|\;M)\right)\;. \end{array}$$ (4)

This shows that perceptuo-motor decoding just consists in a fusion of auditory and motor decoding. This is the way COSMO formalizes, with a minimal set of assumptions, perceptuo-motor theories combining auditory processing with motor predictions [71] or with motor knowledge in the Perception-for-Action-Control Theory that we presented in various papers [73–75].

In a previous paper, we demonstrated that in a set of ideal learning conditions, Eqs (2) and (3) provide actually exactly the same distributions [63]. Ideal conditions mean that (i) sensory learning is perfect (i.e., the Learning Agent perfectly learns the relationship between objects and sensory outputs produced by the Master Agent); (ii) sensory-motor learning is perfect (i.e., the Learning Agent perfectly learns the relationship between gestures and sounds in the environment); and (iii) motor learning is perfect (i.e., the Learning Agent perfectly mimics the motor repertoire of the Master Agent). The fact that in this set of perfect learning conditions Eqs (2) and (3) provide the same output means that in this case the COSMO implementations of the Auditory and Motor Theories become indistinguishable. This is the way COSMO demonstrates in a mathematical way the intuition by tenants of Auditory Theories that all the information provided by the motor system is displayed in the content of the auditory stimuli. Still, we also demonstrated, in the same paper, that in normal conditions where learning is not perfect, the two systems become mathematically distinguishable, and that this leads to a complementarity between auditory and motor decoding. As a matter of fact, auditory decoding appears optimally tuned to the stimuli of the environment, whereas motor decoding appears more able to deal with atypical inputs, such as in a foreign accent or in adverse conditions. This is actually closely in line with neurocognitive data reported in Section “Introduction” [64].

This is where the second hypothesis of this study can be introduced. Indeed, idiosyncrasies in production are a typical case of departure from perfect learning: they are due to the fact that because of the complexity of the learning problem, various agents can select various solutions, differing from one another. In consequence, auditory and motor decoding should differ. The second hypothesis is that while auditory decoding should not display free idiosyncrasies since it is closely linked to the stimuli provided by the Master Agent, motor decoding should mirror motor production and hence display perceptual idiosyncrasies correlated with production idiosyncrasies. Hence, we expect that only a motor or a perceptuo-motor COSMO model of speech perception will feature coupled perceptuo-motor idiosyncrasies, such as observed by [43].

### Implementation and evaluation

#### The Ménard & Schwartz study [43]

The M&S study [43] provides a coherent set of data on correlated idiosyncrasies in production and in perception, which provide a test-bed for the model presented previously and for the evaluation of the two working hypotheses. We will hence present this study in some detail. Their data come from production and perception tasks performed by twelve native French speakers on the ten French oral vowels /i y u e ø o ε œ ɔ a/. Participants were aged between 4 years and 39 years and divided in three age groups in the M&S study, that is, “around 4 years old”, “around 8 years old” and “adults”. This is of little interest in the present work, considering that M&S observed no age effect in terms of idiosyncrasies. Participants had no auditory or articulatory impairment.

The production task simply consisted of 10 repetitions of each of the ten sustained, isolated vowels. From each occurrence, M&S first extracted the values of the first two formants F1 and F2, in Hertz, and then converted them into Barks, considering that the Bark scale better models the natural auditory frequency scale in the human brain than the Hertz scale (*F*[*Bark*] = 7 * asinh(*F*[*Hz*]/650) [76]). Then, they computed, for each vowel category, the mean F1 and F2 values for all the corresponding produced stimuli.

The perception task consisted of an auditory classification test, during which each subject was presented with a set of synthetic stimuli evenly distributed in the formant space matched with the subject’s age, thanks to the use of a specific speech synthesizer integrating a mechanism for generating stimuli adapted to the vocal tract size related to age. Each stimulus was presented once, with all stimuli mixed in a random order. The task was to identify each stimulus as one of the ten vowels listed above (forced-choice identification). For each participant, this resulted in associating to each vowel a set of stimuli categorized in the class corresponding to this vowel. Then M&S defined the perceptual F1 and F2 values of the vowel respectively as the mean of F1 and F2 values in Bark of all the stimuli categorized by the subject as this vowel.

In the next step, they focused on the organization of degrees of vowel height among the ten French vowels which represent phonological contrasts among four degrees of vowel height: high (/i y u/), mid-high (/e ø o/), mid-low (/ε œ ɔ/), and low (/a/). F1 is the major correlate of height, hence they focused on this single parameter, and they measured the distribution of high, mid-high, mid-low, and low vowels along the F1 dimension. To account for differences of vocal tract size associated with sex, age and size of their 12 participants, they normalized the mean F1 value for each mid-high and mid-low vowel by linearly scaling all F1 values, so that the mean value for high vowels /i y u/ was 0 and the mean value for /a/ was 1. This was done separately for the perception and production data. Technically, for a given task (perception or production) and for a given subject, noting *m*_*high*_ the high vowel F1 mean (that is the average of the /i y u/ F1 means) and *m*_*low*_ the low vowel F1 mean (that is the /a/ F1 mean), the relative F1 value for each mid-high or mid-low vowel is given by:

$$Relative\;F1=100*\frac{mean_{vowel}-m_{high}}{m_{low}-m_{high}}.$$ (5)

Therefore, only mid-high and mid-low vowels are kept for analyses, since high and low vowels are used for the data normalization process. The analysis of relative F1 values showed that vowels for each participant were characterized by specific relative values both in perception and in production. To study the correlation between production and perception, M&S grouped couples of produced and perceived F1 measures for each subject and performed a linear regression between produced and perceived values for each of the 6 mid-high or mid-low vowels. This provided significant non-zero correlations, between 0.6 and 0.8, supporting a connection between idiosyncrasies in production and perception. Importantly, the authors checked that the distribution of residues from linear regression in the experimental data did not significantly depart from normality, thus validating the use of linear correlation as a test for relationships between idiosyncrasies in production and in perception.

#### Specification of sensory and motor variables in the VLAM articulatory model

**Fig 3:**
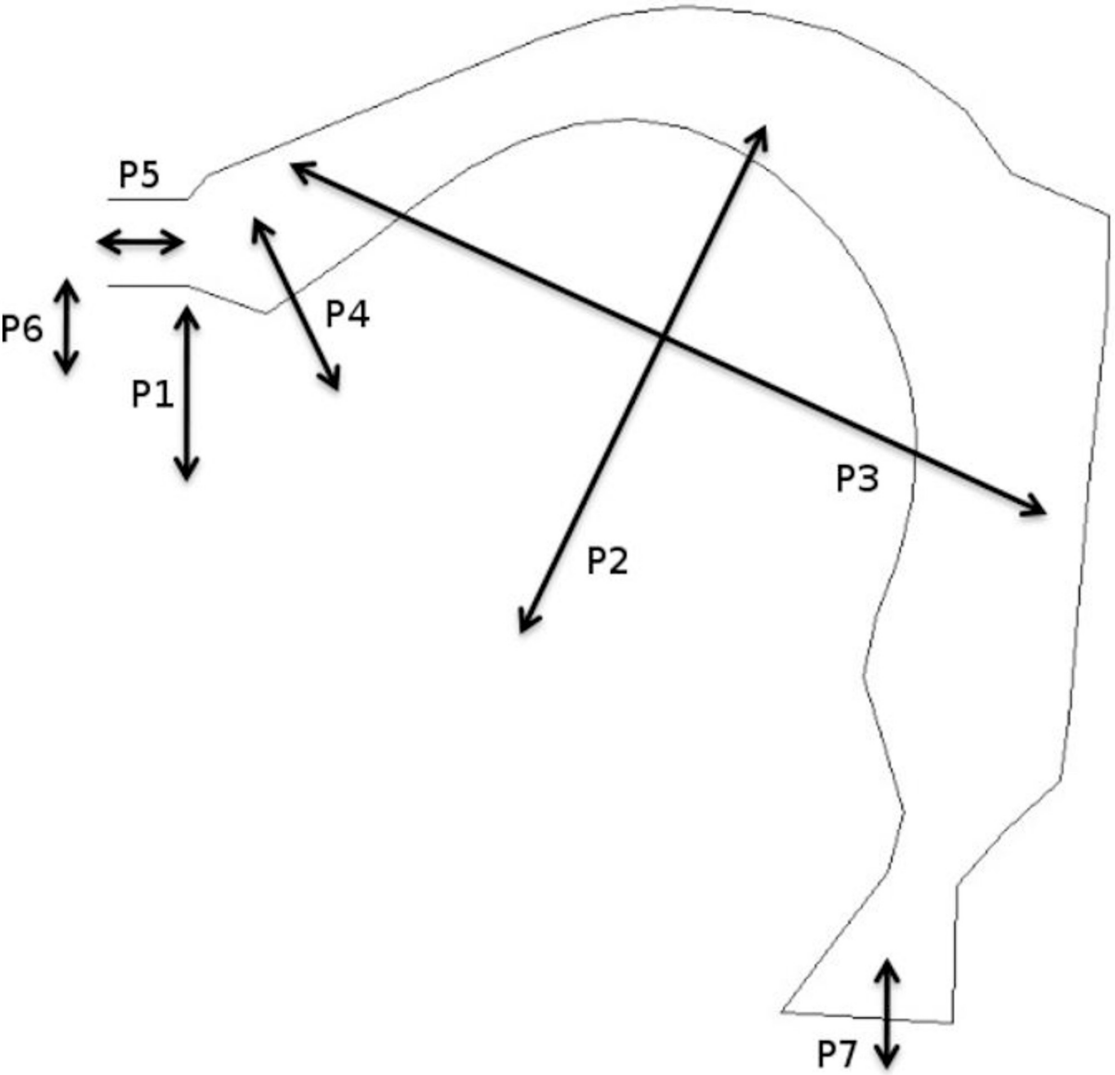
The VLAM model of the vocal tract with its seven articulatory parameters. P1 corresponds to the Jaw parameter, P2 to TongueDorsum, P3 to TongueBody, P4 to Apex, P5 to LipProtrusion, P6 to LipHeight and P7 to Larynx.

In the following, linguistic objects *O*_*S*_ and *O*_*L*_ in COSMO can be one of seven discrete elements corresponding to seven vowels among the ten analyzed by M&S: /i u e o ε ɔ a/, which happen to constitute the preferred 7-vowel system in human languages [77]. We removed the front rounded vowels /y ø œ/ from the analysis to simplify the current study, but we will see that the final results are quite clear and do not seem to depend on the studied vowel. Motor and sensory representations are based on a realistic model of the vocal tract, VLAM (for “Variable Linear Articulatory Model” [78]). This model features seven articulatory parameters derived from a guided Principal Component Analysis of cineradiographic images of the vocal tract. There is one parameter for the position of the jaw (Jaw), one for the position of the larynx (Larynx), three for the shape of the tongue (TongueBody for front/back movement, TongueDorsum for top/down movement, and TongueApex for apex control), and two for the shape of the lips (LipHeight for the closure of the lips, and LipProtrusion for their rounding) (see Fig 3). The production of one configuration of the vocal tract in VLAM provides output formants corresponding to this configuration, that is, the four first peaks in the frequency spectrum of the acoustic signal. In this study, motor gestures *M* are only characterized by three parameters of the model, LipHeight, TongueBody and TongueDorsum, which suffice to produce most values of F1 and F2 in the vocalic triangle (all other parameters being set to zero, that is, a neutral value). The sensory representation *S* corresponds to the F1 and F2 formants. More precisely, after discretization, motor space *M* contains 15,625 articulatory positions (25*25*25) and sensory space *S* contains 4,703 (59*73) couples of formants 〈*F*1, *F*2〉.

#### Specification of the distributions to be learned

The motor repertoire of the Master Agent consists in distributions, which are defined by sets of Gaussian probability distributions—one per object—in the 3D motor space. These distributions are given and fixed in the following way. We define motor vowel prototypes for /i u e o ε ɔ a/, using average formant values for French vowels [79] as targets and selecting values of the three VLAM parameters that best fit the acoustic target. For each vowel, we generated a set of articulatory configurations according to a Gaussian probability distribution centered on the prototype value. The covariance matrix, for each vowel, has 0.1 variance on the diagonal and a 0 covariance value. This ensures that the distributions of the vowels produced by the Master did not overlap. This is of course at odds with what generally occurs in speech production by a natural speaker, but it is part of the set of simplifications introduced in the modeling work to make simulations easier and the logic of interpretation clearer. A drawn motor gesture *m* results in a sound propagated in the environment and received by the Learning Agent as a sound *s*. To simulate a minimal level of noise in the environment, we add a small noise of variance *σ*_*Env*_ to the sound *s* emitted by the Master Agent before reception by the Learning Agent. We have fixed *σ*_*Env*_ to 0.01. Importantly, noise in the environment interferes with the variance of the distributions learned by the agent but not with their means. Hence, the selected value does not have any influence on idiosyncrasies.

The motor repertoire of the Learning Agent consists in the set of distributions *P*(*M* | *O*_*S*_), which are defined by sets of Gaussian probability distributions—one per object—in the 3D motor space. Its sensory repertoire consists in the set of distributions *P*(*S* | *O*_*L*_), which are defined by sets of Gaussian probability distributions—one per object—in the 2D formant space. Its sensory-motor system corresponds to the set of Gaussian probability distributions *P*(*S* | *M*)—one per motor case, that is, 15,625 distributions—in the 2D formant space. The parameters of three sets of distributions are learned according to the method introduced in Section “Learning”.

#### Simulations and evaluation

**Learning** The first stage of our simulations consists in learning distributions of the Learning Agent. This is performed with a fixed Master Agent, defined in the previous section. The Learning Agent also has a fixed structure, as defined in previous sections, with two possible variants in the learning process, which is either “imitative” or “communicative”. For each of these two variants, we perform twelve simulations of the whole learning process, each corresponding to a different Learning Agent learning the same target system provided by the fixed Master Agent. The twelve simulations for each variant differ in their history of learning, that is, in the occurrences of sounds *s* and objects *o* drawn by the Master Agent during the sensory and sensory-motor learning phases, and in the occurrences of gestures *m* and objects *o* drawn by the Learning Agent in the sensory-motor and motor learning phases. Each learning phase (sensory then sensory-motor then motor learning) consists in 300,000 learning steps for each simulation.

**Idiosyncrasies in speech production** Our first evaluation goal is to assess whether simulations with either the imitative or the communicative learning model lead to the emergence of free idiosyncrasies in production. These would be observed if the twelve simulations of a Learning Agent guided by a fixed Master resulted in variability in the distribution of formant values for the learned vowels. Hence, for each simulation and each vowel *o*_*S*_, the Learning Agent produces a set of motor gestures *m* corresponding to object *o*_*S*_ according to its production system *P*(*M* | [*O*_*S*_ = *o*_*S*_]). Then, we observe the sound *s* resulting from motor gesture *m* with the function *s* = *f*(*m*) of the VLAM model. We denote this whole production distribution *P*(*f*(*M*) | *O*_*S*_). We compute its mean in Barks and interpret this mean value as the prototypical value for this vowel. Then, we can compare the positions of vowel prototypes among the 12 Learning Agents for each learning model (imitative or communicative) to evaluate whether they display variations.

For this aim, we evaluate for each learning model and for each vowel the mean distance between the twelve prototypes and the Master prototype (defined in Section “Specification of the distributions to be learned”), characterizing free idiosyncrasies with respect to the Master. The first hypothesis of this study is that these measures would be close to 0 for the imitation model (no idiosyncrasies) whereas the communicative model would produce mean distances significantly larger than 0, resulting from free idiosyncrasies in production [80].

**Idiosyncrasies in speech perception** The second stage of simulations consists in perception tasks performed after the learning stage is ended. The question is to assess whether and how idiosyncrasies in speech perception emerge in the model, and whether they are correlated with idiosyncrasies in speech production. Hence, we only conserve, at the end of the first stage, the learning models that result in idiosyncrasies in production. Then, for each of the twelve simulations of the corresponding learning models, we analyze speech perception tasks as they are modeled by either an auditory theory according to Eq (2), a motor theory according to Eq (3) or a perceptuo-motor theory according to Eq (4).

To define perceptual prototypes in Learning Agents, we proceed in a way similar to M&S. For each vowel and each Learning Agent, we estimate, for each sensory value *s* in the sensory space defined previously, the object *o* selected by a categorization process based on distributions *P*([*O* = *o*] | [*S* = *s*]), where *O* is specified in relation to the considered perception theory: *P*(*O*_*L*_ | *S*) for the auditory model of perception, *P*(*O*_*S*_ | *S*) for the motor model of perception and *P*(*O*_*S*_ | *S* [*C* = 1]), for the perceptuo-motor model of perception. Then, as in M&S, we define the perceptual prototype by the mean of *s* values associated to the object *o* for the corresponding vowel. This results in twelve prototype values for each vowel and each model.

During the analysis, we observe whether these values differ between Learning Agents for one or the other perception model. The second hypothesis of this study is that no idiosyncrasy in the perception task would occur in the auditory model, whereas idiosyncrasies would appear in the motor and perceptuo-motor models. Moreover, they would be correlated to idiosyncrasies in production, since a similar piece of knowledge intervenes in both vowel perception and production.

To assess the existence of such correlations, we focus on the F1 dimension, just as M&S did. The same patterns of results would be obtained for F2 as for F1, but they would not be related to ground truth data. We use the same process as M&S for computing relative F1 values for mid-high and mid-low vowels in both the speech production and speech perception tasks. That is, for speech production and for the three models of speech perception, for each Learning Agent, noting *m*_*high*_ the high vowel F1 mean (which is the average of the /i u/ F1 means) and *m*_*low*_ the low vowel F1 mean (that is the /a/ F1 mean), the relative F1 value for each mid-high or mid-low vowel is given by Eq (5). For each perception model, and for each mid-high and mid-low vowel, we can then compute linear regression between the 12 pairs of values associated to relative F1 values in the production and perception tasks. The second hypothesis predicts that linear regression tests would show correlated idiosyncrasies in perception and production for both the motor and perceptuo-motor models of speech perception.

## Results

### Idiosyncrasies in production: comparing the imitative and communicative learning models

**Fig 4:**
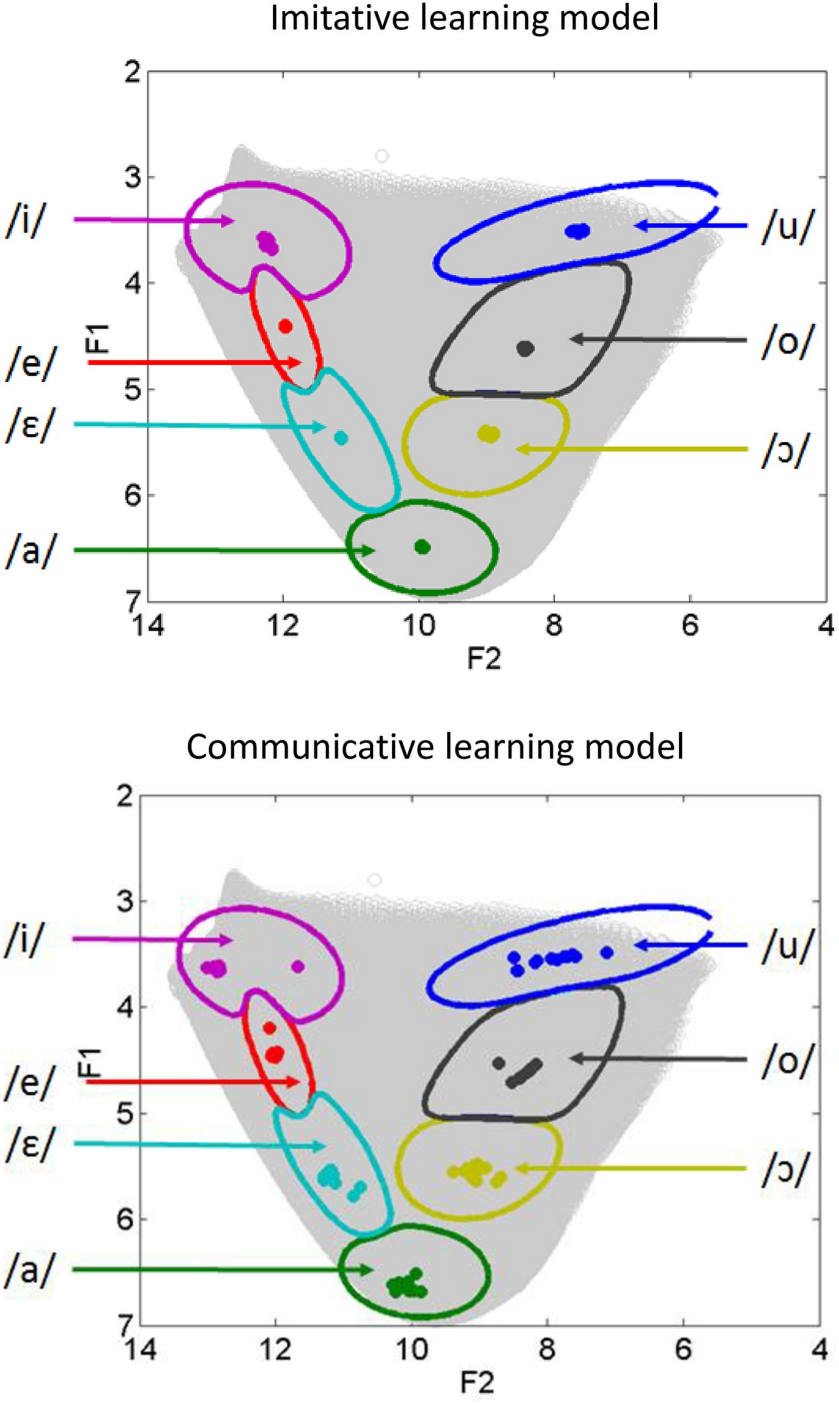
Idiosyncrasies in production after a learning phase involving either the imitative (top) or the communicative (bottom) learning model. Each plot displays vowel prototypes in the speech production task, after learning, for each of the twelve Learning Agents and for each of the seven learned vowels. Vowel prototypes are defined as the mean of the distributions *P*(*f*(*M*) | *O*_*S*_) for each vowel *O*_*S*_. In each plot, prototypes for each vowel and each agent are shown as colored dots in the first two formant acoustic space (F1, F2) (in Barks). To provide a reference, enabling the localization of prototypes in relation to the Master Agent’s vowel space, we also display the *P*(*O* | *S*) distributions for the Master, that is, the categorization regions of the Master Agent for each vowel, computed over all *s* values in the sensory domain (not constrained to be in the vocalic triangle, displayed by the grey area). The categorization regions are visualized as contour lines (contours are drawn one after the other, hence contours drawn later may hide contours drawn sooner).

We first assess the prototypical values obtained at the end of the learning process in the speech production task for the twelve Learning Agents, contrasting the imitative and the communicative learning models. Prototypical values for each agent and each vowel were defined in Section “Simulations and evaluation” (i.e., mean values of the distributions *P*(*f*(*M*) | *O*_*S*_), in Barks). They are shown in the F1-F2 plane in Fig 4. On the top part of Fig 4, we observe that, in the case of imitative learning, the prototypes for the twelve agents during the speech production task have essentially the same values, for each vowel. These prototypes are systematically clustered towards the center of the corresponding categorization region for the Master Agent. Hence, there are basically no idiosyncrasies in the imitative model. In contrast, it appears on the bottom part of Fig 4 that prototypes for the 12 Learning Agents in the communicative learning model are scattered in the acoustic space, although still located in the vowel categorization regions for the Master Agent. This confirms the presence of idiosyncrasies in this latter case.

To quantify idiosyncrasies, we show in Table 1 the mean distances between prototypes for the Learning Agents and for the Master Agent introduced in Section “Simulations and evaluation” for each vowel and each learning model. For the imitation model, mean distances from the individual prototypes to the Master prototypes vary from 0.01 to 0.05 Barks for each vowel, with a mean around 0.03 Bark which is extremely small. This model hence allows to quasi perfectly reproduce the Master prototypes, and generates no free idiosyncrasies.

**Table 1:** Mean distances from the individual prototypes of the 12 Learning Agents to the Master prototypes for both learning models, in Barks. The last line displays the mean values computed over the 7 vowels.

Vowel	Imitative model	Communicative model
/a/	0.01	0.22
/ε/	0.02	0.21
/e/	0.01	0.08
/i/	0.05	0.64
/ɔ/	0.05	0.24
/o/	0.03	0.16
/u/	0.05	0.39
mean	0.03	0.28

For the communicative model, values are 5 to 10 times larger. The mean distance from individual prototypes to the Master prototypes reaches about 0.3 Barks, which is indeed rather large considering that the distances between two neighbor prototypes such as /i/ and /e/ can be less than 1 Bark. This confirms that free idiosyncrasies appear only in the communicative learning model. Importantly, these idiosyncrasies generate individual prototypes systematically inside the correct identification zone (as displayed in Fig 4), which shows that they are phonologically valid.

### Idiosyncrasies in speech perception in relation to speech production: comparing the auditory, motor and perceptuo-motor models of speech perception

In the following, we conserve the only learning model generating free idiosyncrasies in production, that is, the communicative learning model. We use it to simulate the perception task, so as to test the relationship between idiosyncrasies in perception and in production. Hence, we have at our disposal a Master Agent that provided stimuli for learning, and twelve Learning Agents who learned their sensory, sensory-motor and motor distributions from the Master, with variability in motor distributions resulting in the free idiosyncrasies described in the previous section.

**Fig 5:**
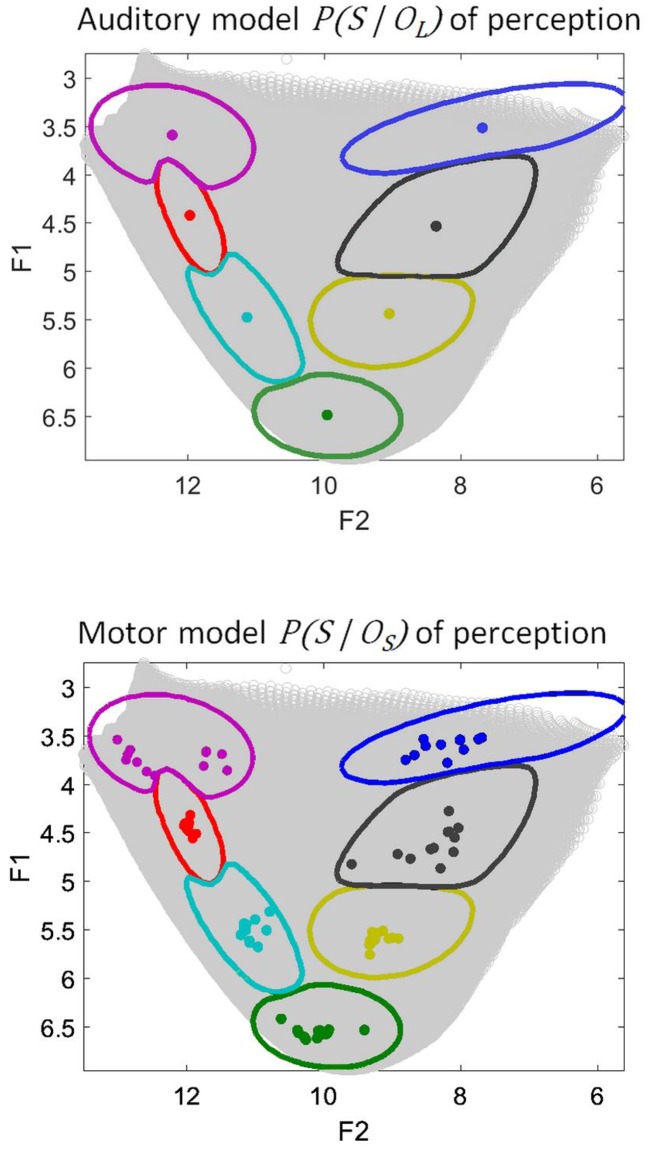
Idiosyncrasies in the auditory (top) and motor (bottom) models of speech perception. Each plot displays prototypes for the auditory or motor decoder, for each of the twelve Learning Agents and for each of the seven learned vowels. Vowel prototypes are defined as the mean of the distributions *P*(*S* | *O*_*L*_) (auditory model) or *P*(*S* | *O*_*S*_) (motor model) for a given Agent and a given vowel *o*. In each plot, prototypes for each vowel and each agent are shown as colored dots in the first two formant acoustic space (F1, F2) (in Barks), following the same presentation as in Fig 4.

We begin by analyzing the behavior of the auditory and the motor models of speech perception. As explained in Section “Implementing auditory, motor and perceptuo-motor theories in COSMO”, they are respectively modeled in COSMO as the answers to the probabilistic questions *P*(*O*_*L*_ | *S*) and *P*(*O*_*S*_ | *S*). For representing these distributions, for each vowel and each Learning Agent, we use the mean value of the distributions *P*(*S* | *O*_*L*_) (Agent prototypes in the auditory model of perception) and of *P*(*S* | *O*_*S*_) (Agent prototypes in the motor model of perception). These prototypes are shown in the (F1, F2) space in Fig 5.

We observe in Fig 5 (top part) that vowel prototypes in the auditory model display values identical for the twelve Agents, exactly at the center of each categorization region, that is, at a value equal to the sensory prototype of the Master Agent. Hence, the auditory model of vowel perception generates no idiosyncrasies. The reason is that the auditory model exploits the *P*(*S* | *O*_*L*_) distribution, which is directly learned from the Master Agent, hence the perceptual prototypes in the auditory model of perception perfectly reproduce the Master prototypes with no variability.

In contrast, the motor model of perception does display perceptual idiosyncrasies (Fig 5, bottom part). As a matter of fact, prototypes are positioned in the acoustic space in a way quite similar to their position in the production task (compare with Fig 4, bottom part). The reason is that both the speech production and the motor model of the speech perception tasks are computed from the same distribution, that is, the motor repertoire *P*(*M* | *O*_*S*_). From this distribution, the production task further involves the transformation of a motor gesture into sound in the environment, through the distribution *P*(*f*(*M*) | *O*_*S*_), whereas the perception task involves the internal model through the distribution *P*(*S* | *O*_*S*_) (see Eq (3)). The similarity of idiosyncrasies in production and in motor perception thus suggests that the internal model of each Learning Agent is a close reproduction of the true articulatory-to-auditory transformation in the environment.

**Fig 6:**
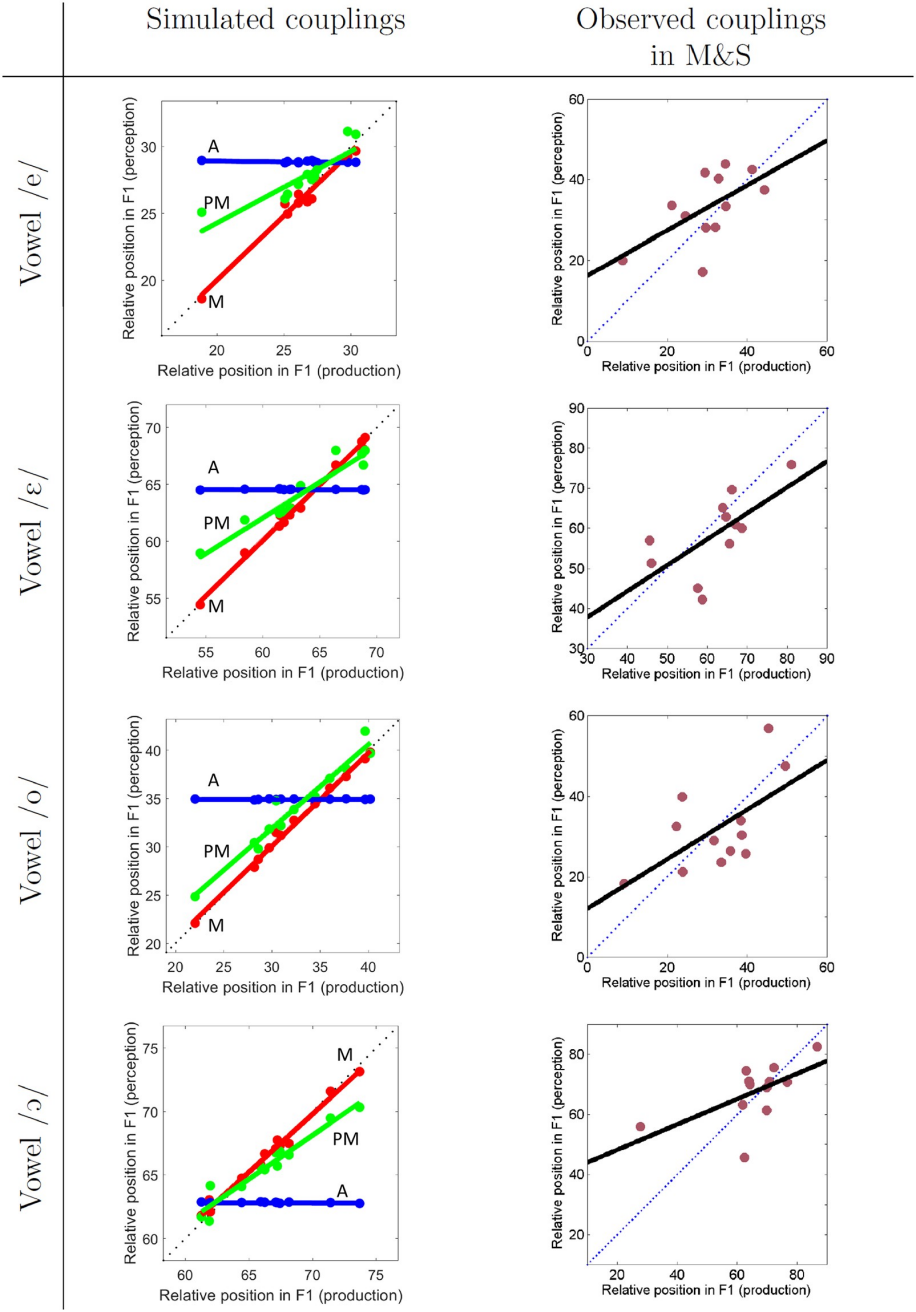
Idiosyncrasies coupling in the auditory, motor and perceptuo-motor models of perception and production. Left column: simulation results and linear regression slopes for the auditory (in blue, A), motor (in red, M) and perceptuo-motor (in green, PM) models. Right column: experimental data points and linear regressions from the M&S study. Each row corresponds to one of the four mid-vowels /e ε o ɔ/.

We then computed relative F1 values, as in the M&S study (see Eq (5)), for all mid-vowels—that are /e, ε, o, ɔ/—both in the production and perception tasks, and considering for the perception task both the auditory, motor and perceptuo-motor models (see Section “Implementing auditory, motor and perceptuo-motor theories in COSMO”). We therefore obtain, for each of the 4 mid-vowels, twelve relative F1 prototype values either in production, or in perception respectively in the auditory, motor and perceptuo-motor models. Fig 6 (left column) displays the perception values relative to the production values in the same manner as in the M&S study (right column). For each vowel and each Learning Agent, simulations provide three points, corresponding to the three perception models, associated to a unique value corresponding to the production task. Three linear regression fits are computed, one for each model, and are also displayed on Fig 6 (left column). Slopes of these regression models are reported in Table 2.

**Table 2:** Slopes of the regression between production and perception data for each mid-vowel in the M&S study compared with slopes of the regression between production and perception simulations in COSMO for the auditory, motor and perceptuo-motor (PM) models of speech perception. We display the significance level of the slope of M&S data with the following format: (superior to 0, inferior to 1). Significance levels are noted * for *p* < 0.05, *o* for *p* ≥ 0.05.

Vowels	M&S	Auditory	Motor	PM
/e/	0.5612 (*, *o*)	-0.0103	1.0097	0.5395
/ε/	0.6505 (*, *o*)	-0.0009	0.9915	0.6290
/o/	0.6195 (*, *o*)	-0.0044	0.9527	0.6898
/ɔ/	0.4236 (*, *)	0.0000	0.9619	0.8685

Slopes for the auditory model are close to 0, since this model yields no perceptual idiosyncrasies. Slopes for the motor model are around 1, since perception in the model is driven by access to motor prototypes through the probabilistic inversion in Eq (3) and slopes for the perceptuo-motor model, which operates a fusion between the auditory and motor models (see Eq (4)), are between 0.5 and 0.9. To compare our simulation results with those of the M&S experimental study (displayed on Fig 6, right column), individual data cannot be directly compared because our simulations do not aim at capturing these specific individual data. Therefore, we focus on global properties of our simulations, that is to say, the regression slopes of the correlations between production and perception data. The comparison between simulations and behavioral data can be summarized in three points.

The first point concerns the extent of idiosyncrasies in production. We observe that simulations yield less idiosyncrasies than there are in behavioral data. Indeed, relative F1 values in production vary by no more than around 14% (from 11 to 18%) in the simulations, whereas they vary by as much as 50% in behavioral data. We will come back on this discrepancy in the next section.

The second point concerns regression slopes of the production-perception link. The slopes vary from 0.4 to 0.6 in behavioral data, and were shown to be significantly higher than 0 (see Table 2). This is clearly incompatible with the auditory model, which results in null slopes in simulations. The slopes in behavioral data are also well under 1, which makes the motor model unlikely. However, statistical tests assessing whether the behavioral slopes are significantly lower than 1 were significant in only one case among the 4 mid-vowels (see Table 2). The simulations of the perceptuo-motor model of speech perception yield regression slopes around 0.6, which are consistent with behavioral data. Altogether, our simulations suggest that a motor component in speech perception, in such conditions, appears necessary to produce perceptual idiosyncrasies correlated with production idiosyncrasies, and that the perceptuo-motor perception model displays correlation slopes which fit well with behavioral data, and better than the purely motor model of perception.

Finally, the correlation coefficients between data in the perception and production tasks (see Table 3) are lower (around 0.60) in the M&S study than in the perceptuo-motor and motor simulations (where they are close to 1). This is of course due to the fact that our simulations are intrinsically less noisy than real data. Indeed, behavioral data are likely to include a large number of factors of interpersonal variation and inter-trial variability, which we did not aim to account for in this study, and were thus not included in the model.

**Table 3:** Correlation coefficients between production and perception data for each mid-vowel for M&S data; together with correlations between production and perception simulations in COSMO in the motor and perceptuo-motor (PM) models of speech perception.

Vowels	M&S	Motor	PM
/e/	0.5965	0.9976	0.8728
/ε/	0.6554	0.9974	0.9573
/o/	0.6153	0.9989	0.9651
/ɔ/	0.6024	0.9987	0.9757

## Discussion

The simulations performed here on vowel production and perception in COSMO aimed at clarifying how free idiosyncrasies in production and in perception could occur in the model, and what are the implications in terms of the nature and role of perceptuo-motor interactions in learning and in speech communication. The simulations confirmed our two principal expectations. Firstly, incorporating a communicative process in the course of learning leads to free idiosyncrasies in vowel production. We will discuss here what the key simulation ingredients are which enable a Learning Agent to converge to actions different from those of its Master, and we will consider limitations of these simulations and possible alternatives.

Secondly, if idiosyncrasies in production are allowed in the model by an adequate communicative learning process, mirror idiosyncrasies emerge in speech perception, provided that a sensory-to-motor link is incorporated in the decoding process, through the use of a motor or perceptuo-motor model of speech perception. Here again, we will address possible limitations and alternatives.

These two discussion parts will shed some light on the two central issues addressed in this paper, that are the nature and underlying mechanisms of speech idiosyncrasies, and the nature and potential functions of perceptuo-motor relationships in speech communication.

### Idiosyncrasies in speech production

#### Generating idiosyncrasies in the communicative learning model

The simulations in Section “Idiosyncrasies in production: comparing the imitative and communicative learning models” first show that no idiosyncrasies emerge when learning is based on a pure imitation of the Master’s productions. In the corresponding model driven by the motor learning question *P*(*M* | *O*_*S*_ *S*), when the sensory-motor model *P*(*S* | *M*) is sufficiently well learned, the agents are able to solve the learning question efficiently. Hence they discover the adequate motor commands, enabling them to carefully reproduce the distribution of sounds associated to the object. Since this distribution is uniquely provided to all agents by the Master, the associated motor productions lead to similar sounds, hence idiosyncrasies are essentially absent.

Conversely, idiosyncrasies do emerge with the communicative learning model driven by the motor learning question *P*(*M* | *O*_*S*_ [*C* = 1]). Let us analyze how such idiosyncrasies can appear in COSMO. The *P*(*f*(*M*) | *O*_*S*_) distribution actually combines two factors, the distribution of the Learning Agent motor system *P*(*M* | *O*_*S*_) and the function *f* corresponding to the transformation of motor gestures *M* into sounds *S*. Of course, since the production task is performed by each Learning Agent in the same environment, the function *f* is identical for all of them, hence idiosyncrasies are actually contained in the motor system *P*(*M* | *O*_*S*_), and more precisely in the way this distribution is learned.

Let us recall that the learning process consists of three steps: sensory, sensory-motor and motor learning. Sensory learning is actually not involved in learning the *P*(*M* | *O*_*S*_) distribution. Sensory-motor learning, based on an imitation process, only serves as an intermediate step for enabling the Agent to learn motor actions associated to sensory results. Therefore, the key ingredient is actually the third learning component, in which the Agent selects adequate gestures *M* for a given object *O*_*S*_. This is performed in COSMO by a communicative process in which the Agent generates a motor gesture *m* drawn according to the *P*(*M* | [*OS* = *o*] [*C* = 1]) distribution, which is computed by Bayesian inference as follows:

$$\begin{array}{l} P(M\;|\;\left[O_{S}=o\right]\;\left[C=1\right])\\ \;\propto \;P(M\;|\;\left[O_{S}=o\right])\\ \;\sum_{S}(P(S\;|\;M)P(\left[O_{L}=o\right]\;|\;S))\;. \end{array}$$ (6)

In this equation, the term

$$\begin{array}{c} A=\sum_{S}(P\left(S\;|\;M\right)P(\left[O_{L}=o\right]\;|\;S)) \end{array}$$

is constant during the third learning phase and forms a distribution likely to be quite similar among Learning Agents since they learned similar *P*(*S* | *M*) and *P*([*O*_*L*_ = *o*] | *S*) distributions. Importantly, the terms *P*([*O*_*L*_ = *o*] | *S*) in this distribution are obtained by the Bayesian inversion of the Gaussian distributions *P*(*S* | [*O*_*L*_ = *o*]), providing plateaus in the motor space that are regions corresponding to one specific object.

These regions of acceptability of each object in the motor space, displayed in Fig 4, provide the basis for the appearance of idiosyncrasies in production. Indeed, contrary to *A*, the other factor in Eq (6), *P*(*M* | [*O*_*S*_ = *o*]), varies during the learning phase and varies between Learning Agents. As a consequence, if a given Learning Agent randomly selects a given *m* value within the acceptable motor plateau at the beginning of the learning process, the *P*(*M* | [*O*_*S*_ = *o*]) term will lead the Agent to keep similar motor actions for the same object in future attempts of communication with the Master, and since the Master will accept these actions as satisfactory, the production process will be anchored around this initial behavior. The *P*(*M* | [*O*_*S*_ = *o*]) term hence leads to the selection of some particular motor gestures in each plateau, differing among the Learning Agents. This creates idiosyncrasies. We noticed in Section “Idiosyncrasies in speech perception in relation to speech production: comparing the auditory, motor and perceptuo-motor models of speech perception” that the range of idiosyncrasies in production was smaller in our simulations than in the experimental data gathered by M&S (see Fig 6). Extending the range of idiosyncrasies could be achieved in the present model by various means, such as increasing the variance of the Master distributions to broaden the width of the regions of acceptable stimuli for each object, or increasing the weight of initial acceptable motor values selected according to Eq (6) to anchor more rapidly the behavior of a given agent at its idiosyncratic initial position.

Idiosyncrasies in this scenario hence emerge from a selection of adequate initial productions as potential solutions for motor learning in excess of degrees of freedom. This is consistent with the view that motor learning exploits generalization processes in which the history of past sensory-motor processes conditions the acquisition of new abilities (see e.g. [81]), and more generally with the large literature on generalization in motor control. It is classical to consider that many developmental idiosyncrasies persist in adulthood. The scenario implemented in the Communicative Learning Model introduces an interesting prediction, that is some specificities of the initial exploration phase in vocal development would result in later characteristics of idiosyncrasies in speech production in childhood and in the later stages of life.

#### One master vs. several masters in the learning process

In these simulations all Learning Agents have the same Master. This simplified and unsatisfactory assumption corresponds to a kind of “pure” situation of free idiosyncrasy emergence. However, we will now analyze how this could be modified and possibly change our conclusion about communication vs. imitation in the learning process. Firstly, each Agent could have a different Master, and hence idiosyncrasies, would appear even with an imitation model, and in any case would simply be reproduced from one generation to the next. This, however, raises two problems: one factual and the other one conceptual. The factual problem is that, according to [39] (see Section “Modeling perceptuo-motor idiosyncrasies to better understand perceptuo-motor relationships in speech communication”), idiosyncrasies are free rather than just reproduced from the environment, since a large part of the idiosyncrasies in vowel production appears to be variable among brothers. The conceptual problem is that, if idiosyncrasies were totally learned, then the problem of idiosyncrasy emergence would be just transferred from one generation to the previous one, and it would remain to explain why the previous generation did display idiosyncrasies.

Another version of the learning model, more realistic, should involve several masters instead of just one. But this would raise typically the same factual and conceptual problems for an imitation learning model. In addition, an imitation model directly applied to a multi-master environment would likely reduce idiosyncrasies from one generation to the next because of the smoothing process associated to the combined statistics of the masters to be learned. It could also be envisioned that imitation in a multi-master environment actually involves a selection process in which one master is selected in the learning process, or a speaker-adaption process in which various masters are learned in parallel [50]. This would actually lead to come back to the variable-master scenario mentioned previously. Altogether, the passage from one master to several seems hence important and relevant from a computational point of view, but it does not modify the analysis about the emergence of free idiosyncrasies.

#### Further analysis of the communicative learning scenario

Interestingly, the communicative model proposed for learning in this study is similar to the communication processes at work in simulations of the emergence of language in societies of interacting agents in a multi-agent scenario [82]. The “interaction games” simulated by e.g., [83, 84] or [85]—and our own simulations of the emergence of phonological inventories in COSMO [58]—are based on various types of interaction situations in which one agent produces a sound and a second agent evaluates the corresponding category and then produces in return an articulatory configuration leading to a sound in the same category. If we make a correspondence respectively between the Master and the Learning Agent in the communicative model here and the two agents in these “interaction games”, the same process is at hand: select a motor gesture to produce a sound corresponding to a given prototype for another agent. All these simulations of interaction games actually lead to the generation of variations in the resulting phonological systems of interacting agents. They can even result in the generation of collective variability within dialects [83]. Therefore, the present simulations with the communicative learning model can be considered an extension of these phylogenetic models to ontogeny.

It is well known that the social context of interaction in the development of speech communication is important (e.g. [67, 68]). An important question in the communication scenario implementation concerns the need for the Learning Agent to actually receive feedback from the Master to be able to develop idiosyncrasies. Indeed, it is increasingly acknowledged that the amount of interaction with caregivers in the environment is actually highly culture-dependent and sometimes highly reduced in some groups and cultures, even though speech learning is performed as well in these conditions [86]. Interestingly, the developmental scenario exploited here bypasses the requirement to receive direct communicative feedback from the Master, since it suffices that the sensory model is sufficiently well tuned to enable the Learning Agent to simulate the phonological knowledge of the Master. As a matter of fact, Eq (6) does show that what intervenes in the computation is actually not the Master feedback, but the *P*(*O*_*L*_ | *S*) function which expresses the Learning Agent model of the Master sensory repertoire. It remains an open (but addressable) problem to study how more sophisticated communication scenarios, including for instance various types of corrective strategies implemented by the Master, could modify and possibly improve the efficiency and adaptability of learning in further COSMO simulations.

This would enable developing these simulations towards more realistic implementations of the speech communication process, replacing the oversimplified (F1, F2) acoustic representations used in this paper by time-frequency cues able to describe all categories of sounds. The challenge, within reach in the COSMO framework, is to progressively develop a computational model able to learn to produce and process natural speech from large sets of examples provided by its environment.

### Idiosyncrasies in speech perception

#### Free vs. induced perceptual idiosyncrasies

The simulations in Section “Idiosyncrasies in speech perception in relation to speech production: comparing the auditory, motor and perceptuo-motor models of speech perception” seem to falsify a pure auditory model of speech perception, at least in the implementation we proposed in Section “Implementing auditory, motor and perceptuo-motor theories in COSMO”, since they generate no perceptual idiosyncrasy. In contrast, the motor and perceptuo-motor models of perception in COSMO do generate perceptual idiosyncrasies, naturally correlated to idiosyncrasies in production, since production and perception are coupled in these two perception models through the sensory-motor connection implemented in the probability questions in Eqs (3) and (4). Quantitatively, predictions by the motor and the perceptuo-motor models of speech perception are different. Indeed, the motor model, with regression slopes close to 1, provides a close to perfect similarity between idiosyncrasies in perception and in production, whereas the perceptuo-motor model only provides a link between produced and perceived idiosyncrasies. As shown in the previous section and in Table 2, the experimental data by M&S rather suggest a link but no perfect equivalence, hence a perceptuo-motor model would appear more likely.

Still, as previously, let us analyze some of the limitations of this study, and discuss possible alternative interpretations. The assumption of a fixed Master for a series of Learning Agents remains of course the first limitation. In the case of idiosyncrasies in speech perception, it could indeed be first proposed that perceptual idiosyncrasies just result from differences in learned environments. As a matter of fact, variations in the Master’s productions would result in variations in the production of Learning Agents, and also, in an auditory theory of speech perception, in correlated variations in their perception. In this reasoning, perceptual idiosyncrasies would be actually induced by the environment, with differences in the environment leading to differences in the specification of perceptual categories. This kind of “idiodialect” in the Master repertoire would result in a similar idiodialect in the Agent repertoire in both production and perception.

A possible counterargument to this reasoning, or at least a hint that part of the correlation between perceptual and motor prototypes is not induced by the environment, comes from a careful analysis of the available experimental data about vowel idiosyncrasies in French. As a matter of fact, in [43], the strength of correlations between produced and perceived vowels for a given speaker was much larger than the correlations obtained in the parallel study of [39]—though on different speakers—about correlations between vowels produced by pairs of brothers. Indeed, correlations between produced and perceived items among individuals were significant for all tested vowels and variability in production explained 42% of the variance in perception for a given subject [43] while correlations between items produced by brothers in a pair were significant for only a third of the tested vowels, and variability in production by a given brother explained only 26% of the variance of the production of the other brother in a pair.

This suggests that the relationships between perceptual and motor prototypes in the study [43] on which the present modeling work is based, were actually free rather than resulting from a simple reproduction of the structure of the phonetic environment. In the following, we will admit the assumption that the coupling between perceptual and motor prototypes is actually related to the “free” component of idiosyncrasies in production. This sets the framework for the continuation of our reasoning hereunder.

#### Associating an endogenous component to exogenous learning to generate perceptual idiosyncrasies

Another interpretation to the existence of a link between free idiosyncrasies in production and perceptual idiosyncrasies mirroring them consists in assuming that Learning Agents could include their own productions in the set of stimuli used to learn the *P*(*S* | *O*_*L*_) distribution. Indeed, the Agent’s utterances provide endogenous stimuli that can be incorporated to exogenous stimuli in the learning set and hence tune the categories around the output of the motor idiosyncrasies. This is actually the interpretation provided by [42]: “Since the voice that one has the most experience with and which one hears most often is one’s own, an individual’s own productions are likely to have an especially important role in the formation of perceptual expectations” (p. 3114).

Of course, it is well known that one hears one’s own voice in a specific way, largely through bone conduction rather that through aerial conduction as with all other speakers. But this does not change the basic characteristics of the spectro-temporal distribution—particularly the formants—and hence there is no reason why this personal knowledge should not be included in the speaker’s distribution. It has actually been shown that not only do we recognize our own voice within specific brain regions associated to self-voice recognition [87, 88], but also that one’s own voice is associated to specific activity in the brain [89, 90] and even that hearing our own voice modulates auditory processes [91, 92]. Therefore, speakers are indeed able to efficiently decode endogenous stimuli.

A way to introduce an endogenous component in the decoding process has been proposed previously, in the framework of a prototypical model of an auditory theory of speech perception, that is the Ideal Adapter model [50]. In this Bayes-optimal model of speech perception, it is proposed that the listener learns from experience the mapping between acoustic stimuli and linguistic categories in relation to the context of the perceptual experience, and a basic component of context consists in the speaker’s identity. The Ideal Adapter includes a variable representing the speaker’s identity, controlling the distribution of sensory stimuli associated to a given phonetic category.

To make this model compatible with the coupled perceptual and motor idiosyncrasies described in M&S, two hypotheses would be necessary. Firstly, it can be envisioned that a Learning Agent in the Ideal Adapter model includes her/his own productions as part of the phonetic experience and hence that the Agent considers her/himself as one of the speaker in the environment. Secondly, it can be assumed that in the case of speech produced by an unknown speaker, or stimuli lacking any cue enabling identification of the speaker, the listener would use her/his own acoustic distribution to model and decode the stimuli to be classified. If we consider that it is actually the case with the synthetic stimuli in M&S, that do not provide information about the speaker, the decoding of these stimuli would indeed be done in reference to the endogenous component in the model. This would result in a correlation between prototypes in production and in perception.

However, basing perceptual decoding on an endogenous set of acoustic stimuli, either directly by mixing endogenous and exogenous sets, or within the multi-speaker framework of the Ideal Adapter, actually raises a theoretical problem for a learning theory. Indeed, the functional role of the *P*(*S* | *O*_*L*_) distribution is to learn the structure of the environment provided by the surrounding speaking agents. Focusing learning on one’s own productions, either by biasing the overall distribution or by using the endogenous distribution as the basis for processing an unknown speaker, would make the recognition process less efficient for dealing with agents different from oneself—which is actually the rule rather than the exception. As a matter of fact, the listener’s own productions have no reason to be a good model of the sounds existing in the environment, and it would probably be more efficient to use an exogenous multi-speaker model rather than a model based on endogenous stimuli.

#### Generating perceptual idiosyncrasies while keeping accuracy for exogenous stimuli in COSMO

Therefore there seems to exist a contradiction between the fact that idiosyncrasies in perception reveal the existence of an endogenous component in speech perception, and the necessity to focus on exogenous rather than endogenous stimuli for efficient decoding. This problem could be solved in COSMO by a property that we have studied in another paper [63]: the “Auditory-Narrow / Motor-Wide” property. We have shown in this paper that the auditory and the motor decoder combined in COSMO in the perceptuo-motor model of perception (see Eq (4)) display an interesting complementarity. According to this property, the auditory decoder is optimally tuned to the acoustic distribution of the speech stimuli in the environment and hence it is more efficient than the motor decoder for decoding clean or typical speech sounds. However, the motor decoder exploits the knowledge of atypical articulatory configurations discovered along the learning process to be able to interpret “exotic” or adverse stimuli (such as in noise or with a foreign accent). Therefore, motor decoding is more efficient than auditory decoding in such cases, and the perceptuo-motor fusion expressed in COSMO by Eq (4) capitalizes on this complementarity and performs more efficiently than both the auditory and the motor perception model for both typical and atypical stimuli.

The “Auditory-Narrow / Motor-Wide” property exploits a crucial characteristic of COSMO, and more generally of a perceptuo-motor model of speech perception. As a matter of fact, not only does it combine an exogenous component—the auditory decoder—with an endogenous component—the motor decoder—but the endogenous component is generative. That is, it has the property to be able to generate stimuli, thanks to the fact that the motor system is both used for motor decoding and for speech production. Generated stimuli can be novel and atypical, especially in early, exploratory stages of learning. These atypical endogenous stimuli can then be exploited to process atypical exogenous stimuli received from the environment. Generativeness hence enables exploration of atypical sensory regions even if they were not represented in a learning phase. This could well be the interest of introducing an endogenous component in the speech perception system.

Another important difference between COSMO and an auditory model involving an endogenous component, à la Ideal Adapter, is that the COSMO approach is *representational*. Indeed, let us consider Eq (4) defining how perceptuo-motor theories are implemented in COSMO. In this equation, the first factor is equal to *P*(*O*_*L*_ | *S*) or proportional to *P*(*S* | *O*_*L*_): this is the distribution corresponding to exogenous stimuli. The second factor, ∑_*M*_ (*P*(*M* | [*O*_*S*_ = *o*])*P*(*S* | *M*)), is equal to *P*(*O*_*S*_ | *S*) and proportional to *P*(*S* | *O*_*S*_): this is precisely the distribution corresponding to endogenous stimuli. However, COSMO has the property to keep the underlying variable *M* represented in this computation, while it would be implicit if only the distribution *P*(*S* | *O*_*S*_) was considered. This representational property is precisely why motor decoding has been initially introduced, in the framework of the Motor Theory of Speech Perception [4, 5] as a way to deal with coarticulation and phonetic invariance.

In conclusion, the correlated idiosyncrasies in production and perception displayed in M&S can be interpreted in three ways. Firstly, they could be just the consequence of differences in the phonetic environment, inducing variations in both the sensory and motor prototypes for a given speaking agent. However, the data by [39] suggest instead that speech idiosyncrasies are largely free. Secondly, the correlated idiosyncrasies could suggest that the speaker’s own productions are introduced in the sensory learning process, either as part of the global multi-speaker distribution, or, in the Ideal Adapter framework, as one specific model possibly intervening in specific decoding contexts. This introduces a novel duality between endogenous and exogenous processes that could be the focus of future studies. Thirdly, the endogenous route could be expanded into a complete generative and representational process in relation to analysis-by-synthesis, hence implementing a sensory-to-motor relationship in the speech perception process. This is the way COSMO analyses the coupled idiosyncrasies displayed by M&S. As shown in this study, we show it is an efficient way to combine endogenous and exogenous processes, making the system robust in both normal and adverse conditions by the “Auditory-Narrow / Motor-Wide” property. Notice that this presents additional benefits in terms of phonetic invariance, related to other complementarities between sensory and motor representations, e.g., plosive place of articulation is better represented in an articulatory/motor space, while vowels are better represented in an auditory space [63].

### Perspectives

This work opens a number of theoretical, computational and experimental perspectives. The major theoretical perspectives deal with the question of endogenous vs. exogenous processes. If there is indeed a specific endogenous processing route, the relationship with motor inference appears naturally. The fact that self-generated speech stimuli can lead to a specific processing gain in the decoding process, not only for sounds but also for seeing the speaker’s lips [93–95] does suggest that the endogenous route is likely mediated by analysis-by-synthesis processes. Data on the role of the somatosensory system in speech perception provide further evidence in this direction [20, 96].

Coming to computational perspectives, data on the self-lipreading advantage [93, 94] suggest that perceptual idiosyncrasies are not only auditory but also visual, and the COSMO model should be expanded towards a multisensory version for dealing with multisensory interactions in speech perception. The connection between COSMO and the Ideal Adapter model is also a perspective, in which the auditory decoder in COSMO could be made more complex for dealing with various masters or interacting agents, and hence opened to a multi-speaker perspective. The underlying questions associated to speaker normalization will also become a computational challenge for COSMO in the future.

Finally, concerning experimental perspectives, the interest of testing hypotheses and simulating experimental data within computational models is that, at the output of the testing and simulation process, we can generate predictions. As already mentioned, previous simulations in COSMO suggest that within a perceptuo-motor model of speech perception, motor knowledge would be more efficient and helpful in adverse conditions—which is in line with experimental data reported in Section “Introduction”. Therefore, we can predict that if perceptual categories were estimated in noise, the motor system would play a larger part in categorization, which would result in an increase in correlations between perception and production idiosyncrasies (i.e., an increase in the slopes displayed in Table 2).

## Conclusion

The present work enabled us to reproduce in the COSMO framework experimental data showing coupled perceptual and motor idiosyncrasies relative to the height dimension in French oral vowels. Idiosyncrasies in production were obtained by the implementation of a communicative process in the learning stage. Idiosyncrasies in perception, coupled with idiosyncrasies in production, are best accounted for by a perceptuo-motor theory of perception, though some alternative interpretations have also been discussed. This set of simulations provides a new argument in favor of the existence of a perceptuo-motor coupling in speech perception. Such a coupling might enable the auditory system to focus on the sounds of the environment in an optimal way, while the motor system would exploit personal production strategies to better deal with stimuli differing from the learned environment, that is, in adverse conditions or for dealing with specific kinds of idiosyncrasies (e.g. speakers with a foreign accent).
